# Influence of an alkalizing supplement on markers of endurance performance using a double-blind placebo-controlled design

**DOI:** 10.1186/1550-2783-9-8

**Published:** 2012-03-20

**Authors:** Daniel P Heil, Erik A Jacobson, Stephanie M Howe

**Affiliations:** 1 Movement Science/Human Performance Lab, Montana State University, H&PE Complex, Hoseaus Room 121, Bozeman, MT 59717, USA

**Keywords:** Cross country skiing, Upper body power, Oxygen uptake, Buffering capacity

## Abstract

**Background:**

Previous research has shown that ingestion of substances that enhance the body's hydrogen ion buffering capacity during high intensity exercise can improve exercise performance. The present study aimed to determine whether the chronic ingestion of an alkalizing supplement, which purports to enhance both intracellular and extracellular buffering capacity, could impact cardiorespiratory and performance markers in trained Nordic skiers.

**Methods:**

Twenty-four skiers (12 men, 12 women), matched for upper body power (UBP), were split into treatment and placebo groups. The treatment group ingested Alka-Myte^®^-based alkalizing tablets (1 tablet/22.7 kg body mass/day) over seven successive days while the placebo group consumed placebo tablets (i.e., no Alka-Myte^®^) at the same dosage. Prior to tablet ingestion (i.e., pre-testing), both groups completed a constant power UBP test, three successive 10-sec UBP tests, and then a 60-sec UBP test. Next, skiers completed the 7-day ingestion of their assigned tablets followed immediately by a repeat of the same UBP tests (i.e., post-testing). Neither the skiers nor the researchers were aware of which tablets were being consumed by either group until after all testing was complete. Dependent measures for analysis included heart rate (HR), oxygen consumption (VO_2_), minute ventilation (V_E_), blood lactate (LA), as well as 10-sec (W10, W) and 60-sec (W60, W) UBP. All data were evaluated using a two-factor multivariate repeated measures ANOVA with planned contrasts for post-hoc testing (alpha = 0.05).

**Results:**

Post-testing cardiorespiratory (HR, VO_2_, V_E_) and LA measures for the treatment group tended to be significantly lower when measured for both constant power and UBP60 tests, while measures of both 10-sec (W10: 229 to 243 W) and 60-sec UBP (W60: 190 to 198 W) were significantly higher (*P *< 0.05). In contrast, there were no significant changes for the placebo group (P > 0.05).

**Conclusions:**

Following the 7-day loading phase of Alka-Myte^®^-based alkalizing tablets, trained Nordic skiers experienced significantly lower cardiorespiratory stress, lower blood lactate responses, and higher UBP measures. Thus, the use of this supplement appeared to impart an ergogenic benefit to the skiers that may be similar to the effects expected from consuming well-studied extracellular buffering agents such as sodium bicarbonate.

## Background

Ergogenic aids are generally described as substances or techniques used to improve athletic performance. Nutrition supplements are often evaluated for their potential as ergogenic aides by testing an athlete's physiological work capacity both before and after consumption of the supplement. For example, numerous studies have tested the efficacy of ingesting sodium bicarbonate or sodium citrate to enhance intracellular and extracellular buffering capacity during high intensity exercise [1-3]. Theoretically, the ingestion of these substances can enhance the body's buffering capacity by absorbing the hydrogen ion (H^+^) by-product from intramuscular ATP hydrolysis, as well as ATP production via sarcoplasmic glycolysis [4]. During high intensity non-steady-state exercise, the rate of H^+ ^ion production exceeds the muscle fiber's ability to buffer and/or remove the H^+ ^ions from the sarcoplasm. As a result, both intracellular and extracellular pH can decrease and subsequently contribute to muscular fatigue [5]. Thus, an enhanced buffering capacity has the potential to ameliorate the impact of increased H^+ ^production on muscular work capacity during exercise.

Recently, an alkalizing nutrition supplement, hereafter referred to as ANS (TAMER Laboratories, Inc., Shorline, WA USA), has been marketed to endurance athletes as a means for maximizing their intracellular and extracellular buffering capacity via a daily mineral-based supplement. According to the manufacturer, regular consumption of this product will supplement the body's ability to buffer excess hydrogen ions resulting from metabolic acidosis during high intensity exercise. As a result, the manufacturer claims that users can expect to experience increased time to fatigue, lower blood lactate levels during steady-state exercise, as well as a more rapid recovery of muscular strength following an intense muscular effort. The supplement comes in tablet form, is consumed at a dose relative to body mass (1 tablet per 22.7 kg), and contains a proprietary blend of ingredients called Alka-Myte^®^. All of the ANS ingredients are allowed by both the U.S. and World anti-doping agencies (i.e., WADA), while Alka-Myte^® ^itself has been granted New Dietary Ingredient (NDI) recognition by the Food and Drug Administration (FDA).

Given the clearance by WADA and the FDA's NDI recognition, it surprising that there are no published controlled studies to evaluate the efficacy of the performance-related claims stated earlier. Therefore, the purpose of this study was to investigate the potential influence of this alkalizing nutrition supplement on previously validated correlates of cross-country skiing performance (i.e., upper body power) [6], as well as cardiorespiratory and blood lactate responses in well-trained competitive Nordic skiers both before and after a 7-day loading period.

## Methods

### Subjects and study design

Competitive Nordic skiers from the surrounding area were recruited to visit the Movement Science/Human Performance Lab on the Montana State University campus on three separate occasions. Competitive skiers familiar with the test protocols used for this study were recruited to help minimize changes expected with athletes performing lab-based performance tests for the first time. All subjects were assigned into a treatment or placebo group, but neither the subjects nor the investigators were aware of the either group's identity until after all data collection was complete (i.e., double-blind placebo-controlled design).

### Procedures

The first visit familiarized subjects with the testing protocol to be used for subsequent visits. Dependent measures recorded during the second visit (i.e., pre-testing) served to establish a baseline for both placebo and treatment groups. Following a 7-day supplement loading phase, the same tests were administered and dependent measures collected during the third visit (i.e., post-testing) and then compared directly to the pre-test measures. Dependent measures of interest included measures of upper body power (UBP), as well as cardiorespiratory and blood lactate responses to the UBP tests.

**Figure 1:**
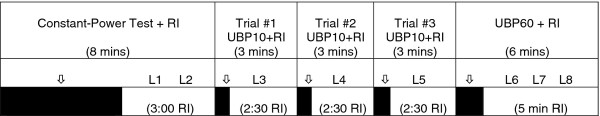
**Diagram of timeline for testing protocol**. The top row shows the order of upper body power (UBP) tests and rest intervals (RI), as well as the total time accumulated (in parentheses) within each measurement period. The second row shows the approximate times at which eight separate fingertip blood lactate samples were collected (indicated sequentially as L1-L8). Arrows within this same row point toward the time period at which the test actually occurred (shown as darkened boxes within third row). Times within parentheses in the third row indicate actual RI time following each test.

During the first visit, subjects read and signed an informed consent document approved by the Montana State University Internal Review Board (IRB). Subjects then practiced with the testing protocols to be used during their second (pre-testing) and third (post-testing) visits to the lab. During the latter two visits, subjects completed a submaximal double poling test (i.e., Constant-Power Test), followed by three trials of a maximal intensity 10-sec upper body power test (UBP10), and then finished with a high intensity 60-sec UBP test (UBP60). An outline of the test protocol administered for both pre- and post-testing is outlined in Figure 1. The third lab visit (i.e., *post-testing*) occurred within 24 hrs of completing the supplement loading phase and repeated all test measures performed during the second lab visit (Figure 1). The first visit lasted approximately 30 minutes, while the other two visits lasted 60 minutes each.

Prior to their pre-testing arrival, subjects were randomly assigned into one of two groups, placebo and treatment, after being matched for their single highest W10 value from the first visit UBP10 tests. For example, the two subjects with the highest UBP10 values were randomly assigned into the placebo and treatment groups, while subsequently ranked pairs were similarly assigned. This group assignment strategy was designed to place skiers with similar caliber of UBP within each test group. The treatment group would consume the ANS tablets while the placebo group would consume placebo tablets during the 7-day loading phase. The ANS tablet manufacturer was able to provide both ANS and placebo tablets (see description below) in sealed packages corresponding to the two groups such that neither the subjects nor the investigators knew the identity of either group.

### Constant-power test

After a 5-minute warm-up on the double poling ergometer at a self-selected power output, subjects were fit with the metabolic measuring equipment and began double poling at a power output equivalent to 50% of the value derived from the UBP10 test (W10, W; from first visit). Using a constant poling cadence, the goal was to reach a plateau in heart rate (HR) and oxygen consumption (VO_2_) within three minutes. The constant-power test continued for 5-mins at which time the poling stopped to draw a fingertip blood sample for the determination of blood lactate. Two blood lactate samples were drawn at approximately 30 and 120 seconds post-exercise (L1 and L2, respectively; Figure 1). Prior to testing, the constant-power test was intended to be a steady-state evaluation of double-poling economy, but the ergometer load (50% of W10) was too high for all subjects to maintain a steady-state over five mins. Thus, the test is referred to as a constant-power test rather than a test of double-poling economy.

### UBP Testing

Immediately following the constant-power test, subjects rested for three minutes before performing three consecutive trials of the UBP10 test. The 10-second test protocol is imbedded within a 30-second time period where the skier spends the first 20 seconds ramping up power output and poling cadence before exerting a maximal double poling effort the final 10 seconds. The average power output measured by the ergometer during the final 10 seconds is recorded as the 10-second UBP (W10, W). Each 30-sec test period was followed by 2.5 mins of rest prior to beginning the next 30-sec UBP10 test period. Subjects used the first trial as an additional warm-up, using approximately 80% of maximal effort during the last 10 seconds, before giving 100% effort for the final two trials. Next, subjects rested again for an additional 2.5 mins before performing a single 60-sec test during which the goal was to achieve the *highest average power output *over the entire 60 seconds (W60, W) when starting from a dead stop. Thus, dependent measures of UBP from these tests included both W10 (best of the last two of three trials) and W60 (one trial only). During the UBP testing, the metabolic measurement system was continuously measuring both HR and VO_2_, while recovery measures of fingertip blood lactate were measured at 30 and 120 secs immediate post-exercise into each rest interval.

Previous research in our lab has determined that measures of both W10 and W60 correlate highly (r ≥ 0.92) with 10 km classical Nordic ski race performance [6]. At 10 seconds of maximal effort, the UBP10 test was designed to emphasize utilization of the ATP-PCr energy system, whereas the UBP60 test was designed to emphasize use of the glycolytic system. Thus, the basis for using the W10 and W60 measures within the current study is the supposition that any factor, such as a nutrition supplement, that can influence measures of W10 and/or W60 could potentially influence actual Nordic ski racing performance as well.

Additional research in our lab has established reliability characteristics for the W10 and W60 measures (i.e., day-to-day repeatability). A local group of competitive Nordic skiers, each with 3+ years of ski racing experience, participated in two UBP testing visits in our lab within 24 hours to two weeks of each other. During each test visit, the UBP10 and UBP60 tests were administered exactly as described for the present study. Specifically, three UBP10 tests were followed by a single UBP60 test with a fixed amount of rest between tests. Subjects who had never performed these tests prior to the reliability study returned for a third visit (i.e., first visit data were not used for data analysis). Mean values for W10 and W60 across the first (Mean ± SE: 208 ± 21 W and 164 ± 16 W, respectively) and the second tests (210 ± 22 W and 162 ± 16 W, respectively) did not differ significantly (*P *= 0.55 and 0.39, respectively). In addition, intraclass correlations, whether computed across two days of testing (ICC > 0.99) or extrapolated for a single measurement (ICC > 0.98), were high, while the standard errors of measurement for both W10 (± 2.7 W) and W60 (± 2.0 W) were low. Collectively, these data indicate that the UBP10 and UBP60 test variables were reliable when using trained Nordic skiers familiar with the test protocols.

### Nutrition supplement

The treatment group consumed ANS tablets at the prescribed dosage of 1 tablet per 22.7 kg (i.e., 50 lbs) of body mass per day, with half to two-thirds of the dosage consumed in the morning and the remainder at night before going to bed. The ANS tablets are considered a mineral supplement with each tablet containing calcium (225 mg), magnesium (1 mg), potassium (36 mg), in a proprietary blend of ingredients called Alka-Myte^® ^(1000 mg). According to the manufacturer, there have been no significant adverse events reported to acute or chronic consumption of this supplement. The placebo tablets were formulated by the ANS manufacturer to have a similar size, color, shape, and texture as the ANS tablets while lacking the Alka-Myte^® ^active ingredient. Those subjects assigned to the placebo group consumed a placebo tablet (maltodextrin-based) in the same dose (1 tablet/22.7 kg body mass/day), frequency (split over morning and evening), and duration (7 day ingestion period) as prescribed for the treatment groups' consumption of ANS tablets.

### Instrumentation

#### UBP Ergometer

A modified Concept 2 rowing ergometer (Concept 2 Model D; Morrisville, VT, USA), similar to that described by Nilsson et al. [7], was used for all UBP testing in this study. In place of the sliding seat on a typical Concept 2 ergometer, a resistance-loaded trolley is connected to the chain that turned the air-braked flywheel. Two cross-country ski poles are attached to the trolley such that pushing on the poles slides the trolley backward along the rail. The chain, in turn, spins the ergometer's flywheel which thus provides resistance each time the poles are pushed backward. As the poles are brought back forward during the recovery phase, the trolley is pulled forward by the pole tips along the rail with very little resistance. Additional ergometer modifications included a longer steel rail than a typical rowing ergometer (2.8 m instead of 1.4 m), as well as a platform mounted above and to the front of the rail on which the skier stands during testing. Identical to the Concept 2 ergometer, the modified ergometer provides a continuous digital display of power output in watts (using the Concept 2 PM3 digital monitor), as well as a recording of average power output over user-defined work periods. Previous research has reported the test-retest of power measurements using the Concept 2 ergometer to have been reliable in tests lasting 90 to 420 seconds [8]. The ski poles used for ergometer testing (Toko P232 poles; Mammut Sports Group AG, Seon, Switzerland; Swix synthetic cork grips and Swix Pro Fit straps; Swix Sport USA Inc., Boston, MA), available in 5-centimeter increments between 135 cm and 170 cm, were fit to within 2.5 cm of each subject's own classic racing pole length. The length of poles used by each subject during the first visit was recorded and used for testing during each subsequent visit.

#### Indirect calorimetry

Based upon unreported pilot testing prior to this study, we speculated that one or more cardiorespiratory parameters could be influenced by ANS if there was a subsequent influence on measures of UBP. Thus, measures of oxygen consumption (VO_2_, L/min), minute ventilation (V_E_, L/min), and heart rate (BPM) were incorporated into the protocol. Using standard indirect calorimetry procedures, a portable metabolic system (Oxycon Mobile, Viasys Healthcare, Yorba Linda, CA) was worn by each subject using a modified hydration backpack (Slipstream; Camelbak Products, LLC; Petaluma, CA). The oxygen and carbon dioxide analyzers were calibrated prior to each test using a certified gas mixture. Both analyzers, as well as the ventilation meter, were calibrated prior to each test according to the manufacturer's guidelines. The metabolic system collected breath-by-breath data which was then reported as 60-sec (for the Constant-Power Test) and 5-sec sample intervals (UBP10 and UBP60 tests) for both VO_2 _and V_E_. Using a Polar Accurex Plus heart rate monitor strap (Polar Electro, Inc., Lake Success, NY), the metabolic system also collected and reported heart rate (BPM) data over the same 60- and 5-sec intervals. During testing, the raw data signals from the metabolic system, including that for HR, were transmitted via telemetry to a computer base station within 20 meters of the UBP ergometer (telemetry range is < 1000 meters).

#### Blood lactate analyzer

Using the handheld Lactate Pro analyzer (Arkray, Inc., Kyoto, Japan), whole blood lactate from a single fingertip blood droplet is analyzed in 60 seconds. The reagent test strip for the meter requires 5 μl of whole blood, sampled by capillary action, to initiate an internal chemical reaction and subsequent electrical current proportional to the lactate concentration. Previous research has shown that while correlations between blood lactate values from different analyzers using the same blood sample can be high (r ≥ 0.97), the absolute difference between monitors can be practically meaningful (± 2-3 mM) over the physiological range of 1-18 mM). To help control for known confounders to the measurement of blood lactate for this study, several precautions were taken. First, the monitor's Check Strip (allows a self-check by the monitor) and Calibration Strip (comes with each box of reagent strips) was utilized prior to each test session. Second, it is known that lactate concentrations can vary between boxes of test strips for the same blood sample. To help control for this variability, a single box of test strips was assigned to each subject for both pre- and post-testing lactate measures. Third, fingertip sampling for blood lactate can be highly variable due to inconsistent skin cleaning and sampling procedures. Lastly, it is possible for individual Lactate Pro meters to provide slightly variable lactate measures for the same blood sample. These last two factors were mitigated with standard measurement protocols, using the same researcher for all lactate testing and by using the same meter for each subject for both pre- and post-testing. Given the controls mentioned above, Pyne et al. [9] concluded that "the Lactate Pro is accurate, reliable and exhibits a high degree of agreement with other lactate analyzers".

#### Diet and exercise log

Both diet and recent exercise habits could confound the measures of UBP, as well as the cardiorespiratory and blood lactate responses by influencing intracellular and/or extracellular buffering capacity. To address this issue, we attempted to control these factors within each subject rather than across all subjects. Using a simple 2-page diet and exercise log, subjects recorded the general types and amounts of food consumed during the 48 hrs preceding testing. Subjects also used the log to record the types of exercise (mode, intensity, duration) in which they participated during the same time period. Subjects were asked to refrain from high intensity and long duration activities for the 24 hrs preceding both pre- and post-testing. After an evaluation of the log by researchers at the end of the pre-testing visit, subjects kept the logs for reference during the 48 hrs prior to the post-testing visit. Ideally, subjects were to use the 2-page log as a reference so that their diet and activity habits were relatively similar prior to pre- and post-testing lab visits. An additional 2-page log was maintained for both diet and exercise for the 48 hrs prior to post-testing. At the end of the post-testing visit, the log was again reviewed by researchers to verify what was recorded. Analyses were not performed on the nutrition and exercise log data, but rather used as a method to assist subjects with adhering to the requirements of the study.

Lastly, subjects were asked to use the 2-day logs as a means for recording any perceived side effects of ingesting the placebo or ANS tablets. Subjects were instructed to consider unusual or unexpected gastrointestinal (GI) distress (e.g., stomach aching or cramping, excess gas), or any other unusual physiological sensations, as possible side effects.

### Statistical analyses

Summary measures of power output (W10, W60), cardiorespiratory measures from the constant-power test (60-sec HR, VO_2_, V_E_), peak cardiorespiratory measures from the UBP10 and UBP60 tests (5-sec HR, VO_2_, V_E_), as well as recovery blood lactate measures following each test (L1-L8) were evaluated using multivariate two-factor (group × time) repeated measures analysis of variance (ANOVA). Post-hoc testing was performed using planned contrasts to compare pre-testing and post-testing values within placebo and treatment groups (alpha = 0.05). Using the procedures described by Cohen [10] and the UBP reliability reported by previously [6], a sample size of 10-12 subjects per group were needed to detect a mean difference of 10-15 W (Power = 0.80 and alpha = 0.05).

## Results

A total of 26 subjects were recruited but only 24 were able to complete all three lab visits. The demographics for the remaining 24 subjects (12 per group), summarized in Table 1 show an equal representation of men and women. This latter characteristic of the two groups was not planned apriori, but rather the result of the W10 matching and splitting strategy. All subjects had been Nordic ski racing between five and 20 years with all but one subject training and competing in Nordic ski races during the recently completed ski season. The 2-day diet and exercise logs for both pre- and post-testing were collected from all subjects. According the subjects, the act of recording diet and exercise habits prior to pre-testing was useful for monitoring and controlling these behaviors prior to the post-testing visit. Lastly, reports of perceived side effects were relatively minimal. Four subjects within the placebo group reported usual GI disturbances (upset stomach over 7 days; unusual gas over 2 days) or events (bad taste to capsules; unusual color in urine and feces noted), while only one subject in the treatment group noted unusual bowel movement activity while ingesting the ANS tablets. None of these perceived side effects, however, were reported to have limited or changed anything about the affected subjects' usual diet or exercise habits.

**Table 1:** Descriptive statistics for demographic variables corresponding to placebo and treatment groups

Group	Gender	Sample Size	Age (years)	Body Height (cm)	Body Mass (kg)
Placebo	Men	7	29 ± 9 (20-47)	178.5 ± 7.8 (167.1-188.5)	76.9 ± 8.8 (66.1-90.5)
(n = 12)	Women	5	29 ± 11 (18-44)	167.6 ± 4.6 (162.4-171.5)	61.3 ± 8.5 (52.4-75.0)
Treatment	Men	7	27 ± 12 (19-52)	180.6 ± 9.1 (169.2-195.0)	72.7 ± 3.4 (68.5-78.2)
(n = 12)	Women	5	21 ± 3 (18-26)	167.8 ± 4.7 (163.3-175.1)	63.7 ± 5.3 (57.6-70.7)

NOTE: All values expressed as Mean ± SD (Range)

### Measures of UBP

**Figure 2:**
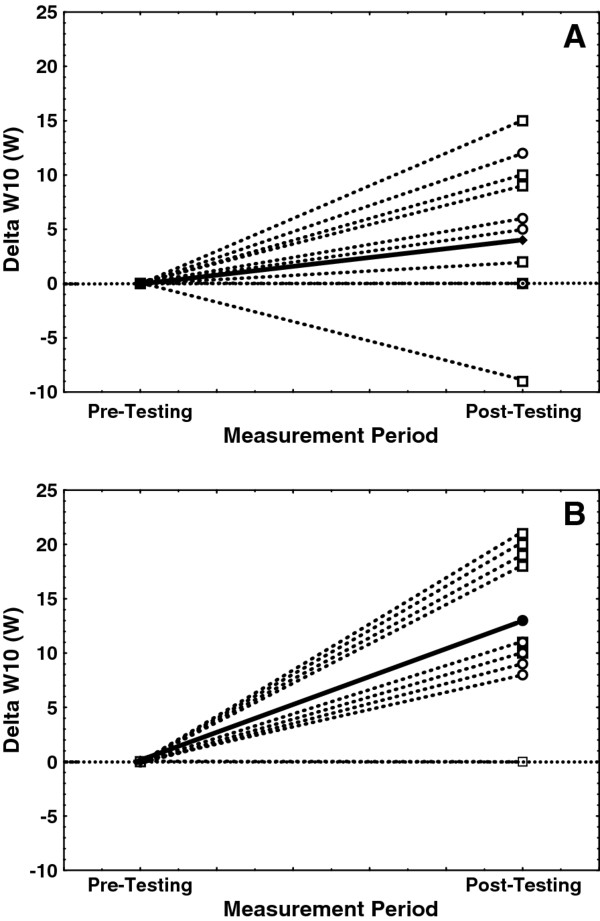
**Individual changes in 10-sec upper body power (Delta W10, W)**. These data represent measured changes following a 7-day nutrition supplement loading period (pre- versus post-testing) for both placebo **(A) **and treatment **(B) **groups. Note that values for men are indicated with dashed lines and open squares (□), women by dashed lines and open circles (○), and change in the group mean is indicated with a solid line and closed diamond (♦). The horizontal dotted line indicates no change between pre- and post-testing.

**Figure 3:**
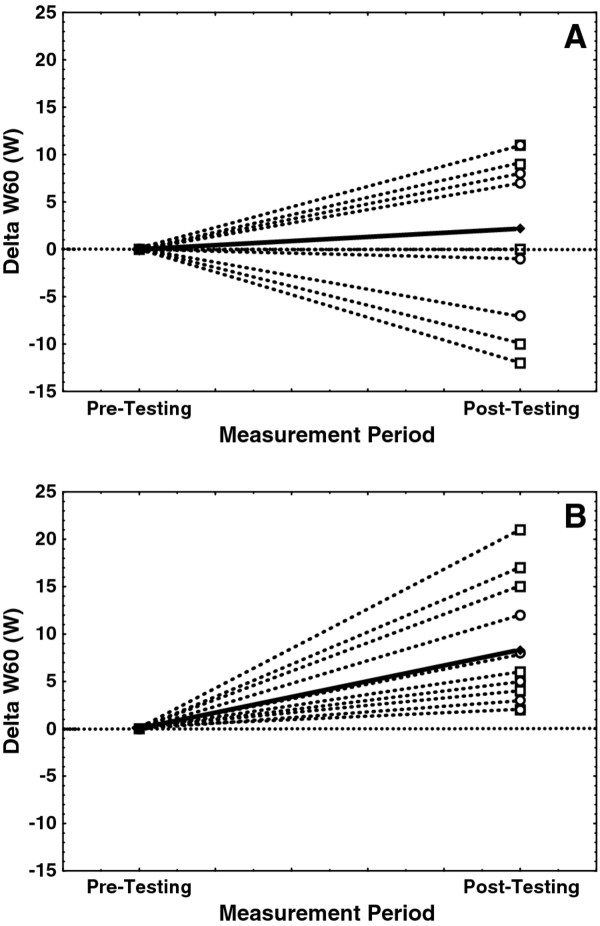
**Individual changes in 60-sec upper body power (Delta W60, W)**. These data represent measured changes following a 7-day nutrition supplement loading period (pre- versus post-testing) for both placebo **(A) **and treatment **(B) **groups. Note that values for men are indicated with dashed lines and open squares (□), women by dashed lines and open circles (○), and change in the group mean is indicated with a solid line and closed diamond (♦). The horizontal dotted line indicates no change between pre- and post-testing.

Mean values for W10 and W60 across test groups and UBP tests (Tables 2 and 3, respectively) show that W60 values were approximately 75-85% of the W10 values, an observation consistent with previous W10 and W60 testing in collegiate Nordic skiers [6]. Mean W10 values for the placebo group were statistically similar across familiarization, pre-testing, and post-testing trials (241-250 W; Table 2). Similarly, W60 for the placebo group did not differ significantly across the three lab visits (186-188 W; Table 3). In contrast, post-testing values for both W10 (Table 2) and W60 (Table 3) were significantly higher for the treatment group relative to familiarization and pre-testing values. Post-testing W10 values were +14 W higher than pre-testing values for the treatment group compared with only +4 W higher for the placebo group. Similarly, post-testing W60 values were +8 W higher than pre-testing values for the treatment group compared with only +2 W for the placebo group. Figures 2 and 3 illustrate the range of individual changes in W10 and W60, respectively, from pre- to post-testing for both placebo and treatment groups.

**Table 2:** Summary of 10-sec upper body power (W10) results for familiarization, pre-testing, and post-testing visits

Group	Best W10 for the *Familiarization* Trial (W)	Best W10 for the *Pre-Testing* Trials (W)	Best W10 for the *Post-Testing* Trials (W)
Placebo (n = 12)	241 ± 30	246 ± 31	250 ± 31
Treatment (n = 12)	225 ± 28	229 ± 29	†243 ± 29

NOTE: All values expressed as Mean ± SE.

†Mean post-testing W10 for the treatment group was significantly higher than the treatment groups' mean values for familiarization and pre-testing.

**Table 3:** Summary of 60-sec upper body power (W60) results for familiarization, pre-testing, and post-testing visits

Group	W60 for the *Familiarization* Trial (W)	W60 for *Pre-Testing* Trial (W)	W60 for *Post-Testing* Trial (W)
Placebo (n = 12)	187 ± 24	186 ± 23	188 ± 22
Treatment (n = 12)	188 ± 22	190 ± 24	†198 ± 25

NOTE: All values expressed as Mean ± SE

†Mean post-testing W60 for the treatment group was significantly higher than the treatment groups' mean values for familiarization and pre-testing

### Cardiorespiratory measures

Summary statistics for measures of HR, VO_2_, and V_E _are presented in Tables 4, 5, 6, respectively. Pre- to post-testing mean HR, VO_2_, and V_E _values for the placebo group were statistically similar across the UBP tests. The one exception was mean post-testing VO_2 _for the UBP60 test which was significantly higher than the placebo group's pre-testing value. Similarly, cardiorespiratory measures for the treatment group did not different significantly between pre- and post-testing conditions for all three trials of the UBP10 test. However, post-testing HR and VO_2 _were both significantly lower than pre-testing values for the treatment group's constant-power test. Additionally, all post-testing cardiorespiratory variables (HR, VO_2_, and V_E_) for the UBP60 test were significantly lower than the group's pre-testing values.

**Table 4:** Summary *heart rate *(BPM) measures resulting from five upper body power tests

Group	Test Period	Constant Power Test	UBP10 Test Trial #1	UBP10 Test Trial #2	UBP10 Test Trial #3	UBP60 Test
Placebo (n = 12)	Pre-Test	161 ± 4	156 ± 2	167 ± 2	169 ± 2	182 ± 2
	
	Post-Test	160 ± 4	157 ± 3	167 ± 3	169 ± 3	183 ± 2

Treatment (n = 12)	Pre-Test	164 ± 6	158 ± 5	165 ± 4	166 ± 4	184 ± 4
	
	Post-Test	†159 ± 6	160 ± 6	169 ± 6	167 ± 4	†177 ± 3

NOTE: All values expressed as Mean ± SE; UBP10 = 10-sec upper body power test; UBP60 = 60-sec upper body power test

†Post-test measure of heart rate for Constant-Power and UBP60 tests were significantly lower than pre-test measure within the treatment group (*P *< 0.05)

**Table 5:** Summary *oxygen consumption *(VO_2_, L/min) measures resulting from five upper body power tests

Group	Test Period	Constant Power Test	UBP10 Test Trial #1	UBP10 Test Trial #2	UBP10 Test Trial #3	UBP60 Test
Placebo (n = 12)	Pre-Test	2.68 ± 0.28	2.29 ± 0.22	2.55 ± 0.20	2.63 ± 0.19	3.14 ± 0.24
	
	Post-Test	2.72 ± 0.30	2.47 ± 0.23	2.88 ± 0.23	2.74 ± 0.21	‡3.38 ± 0.26

Treatment (n = 12)	Pre-Test	2.84 ± 0.29	2.34 ± 0.18	2.61 ± 0.17	2.65 ± 0.19	3.43 ± 0.25
	
	Post-Test	†2.77 ± 0.28	2.33 ± 0.21	2.67 ± 0.23	2.68 ± 0.20	‡3.26 ± 0.26

NOTE: All values expressed as Mean ± SE; UBP10 = 10-sec upper body power test; UBP60 = 60-sec upper body power test

†Post-test measure of VO_2 _for Constant -Power test was nearly significantly lower than pre-test measure within the treatment group (*P *< 0.10)

‡Post-test measure of VO_2 _for UBP60 test were significantly different than pre-test measure within the corresponding test group (*P *< 0.05)

**Table 6:** Summary *minute ventilation *(V_E_, L/min) measures resulting from five upper body power tests

Group	Test Period	Constant Power Test	UBP10 Test Trial #1	UBP10 Test Trial #2	UBP10 Test Trial #3	UBP60 Test
Placebo (n = 12)	Pre-Test	100.6 ± 11.5	87.9 ± 10.0	99.4 ± 10.2	110.4 ± 9.4	147.2 ± 12.1
	
	Post-Test	100.7 ± 13.2	92.3 ± 9.9	109.8 ± 9.9	113.9 ± 10.1	148.6 ± 13.2

Treatment (n = 12)	Pre-Test	104.6 ± 11.7	102.9 ± 7.7	126.0 ± 12.8	129.7 ± 10.3	163.5 ± 12.0
	
	Post-Test	101.7 ± 10.6	97.3 ± 9.8	122.4 ± 11.8	132.1 ± 12.9	†153.3 ± 11.1

NOTE: All values expressed as Mean ± SE; UBP10 = 10-sec upper body power test; UBP60 = 60-sec upper body power test

†Post-test measure of V_E _for UPB60 test was nearly significantly lower than pre-test measures within the treatment group (*P *< 0.10)

### Blood lactate measures

Summary statistics for blood lactate measured at eight separate time points (L1-L8) are shown in Table 7. Pre- and post-testing lactate values were statistically similar for the first seven time points for the placebo group. The placebo group's post-testing value for the eighth time (L8) point was significantly higher than the pre-testing value (10.3 ± 0.6 vs 9.7 ± 0.6 mmol/L). Pre- and post-testing values for the treatment group were also statistically similar for L2-L6, but post-testing values for L1, L7, and L8 were all statistically lower than their corresponding pre-testing values.

**Table 7:** Summary measures for eight (L1-L8) fingertip blood lactate analyses (mmol/L) from tests of upper body power

	Constant-Power Test		UBP10 Tests			UBP60 Test		
**Group**	**†L1**	**L2**	**Trial #1** **L3**	**Trial #2** **L4**	**Trial #3** **L5**	**L6**	**L7**	**L8**

Placebo Pre-Testing Post-Testing	5.3 ± 0.6 4.9 ± 0.6	5.0 ± 0.6 5.0 ± 0.7	5.1 ± 0.6 4.9 ± 0.6	5.6 ± 0.5 5.6 ± 0.5	6.4 ± 0.6 6.6 ± 0.5	9.2 ± 0.5 9.4 ± 0.5	9.8 ± 0.6 10.3 ± 0.6	9.7 ± 0.6 ‡10.3 ± 0.6

Treatment Pre-Testing Post-Testing	7.0 ± 0.6 ‡5.5 ± 0.6	5.8 ± 0.8 5.7 ± 0.7	6.1 ± 0.7 5.6 ± 0.7	6.6 ± 0.6 6.3 ± 0.6	7.2 ± 0.6 7.2 ± 0.7	9.8 ± 0.8 9.4 ± 0.7	10.9 ± 0.7 ‡9.8 ± 0.7	10.4 ± 0.7 ‡9.4 ± 0.6

NOTE: All values expressed as Mean ± SE; UBP10 = 10-sec upper body power test; UBP60 = 60-sec upper body power test. Sample sizes remained at 12 subjects per group for both pre- and post-testing

†The eight blood lactate samples (L1-L8) are labeled the same as those shown within Figure 1

‡Mean post-testing blood lactate value differed significantly (*P *< 0.05) from corresponding pre-testing value within the same test group

## Discussion

The present study was designed to evaluate the potential influence of an Alka-Myte^®^-based alkalizing nutrition supplement (ANS) on cardiorespiratory, blood lactate, and upper body power (UBP) measures in trained Nordic skiers. Collectively, the results from the constant-power and UBP60 tests suggest that, in comparison to ingesting the placebo, a 7-day supplement loading period imparted what could be interpreted as an ergogenic effect on several dependent variables for two of the three tests administered. For example, post-testing cardiorespiratory (HR, VO_2_, V_E_) and blood lactate values tended to be lower for both constant-power and UBP60 tests while the ability to generate power over 60-seconds (i.e., W60 values) was significantly higher following ANS supplementation. In contrast, results from the UBP10 tests provided a less definitive ergogenic effect for the treatment group despite the fact that the treatment group experienced a significant increase in W10 over the placebo group's lack of significant change.

### Constant-power test

The constant-power test involved double-poling on the ergometer for five minutes at an UBP equivalent to 50% of W10. This test was originally intended to elicit steady-state cardiorespiratory and blood lactate responses and thus provide measures of moderate-high intensity double-poling economy. In fact, more than half of the subjects showed small but steady increases in cardiorespiratory parameters during the last 2-3 minutes of the test (i.e., non-steady-state responses). The subjects most likely to experience non-steady-state responses to the protocol, which were evenly split across placebo and treatment groups, were those with the highest W10 values. Regardless, the treatment group's change from pre-testing to post-testing for HR (164 to 159 BPM), VO_2 _(2.84 to 2.77 L/min), blood lactate (7.0 to 5.5 mmol/L) were all significantly lower for the constant-power test whereas significant changes for the placebo group were not observed. This suggests that the treatment group experienced less cardiorespiratory and/or muscular stress while double-poling at the same mean power output following seven days of ANS supplementation.

Interestingly, the above observations are similar to previous evaluations of the influence of pre-exercise metabolic alkalinization on VO_2 _kinetics [11-13]. For example, Kolkhorst et al. [12], using pre-exercise sodium bicarbonate (NaHCO_3_) ingestion to induce metabolic alkalosis prior to high intensity exercise, found that the rapid component of VO_2 _kinetics was slowed when compared to the control condition. Berger et al. [11] also found pre-exercise NaHCO_3 _ingestion to influence VO_2 _kinetics during high intensity exercise, but they also found that end-exercise VO_2 _was significantly lower (2.79 versus 2.88 L/min) at the end of six minutes of high intensity exercise when compared to the control condition. The present study observed a similar decrease in VO_2 _(2.84 to 2.77 L/min; Table 5) with a concomitant decreases in HR (164 to 159 BPM; Table 4) and blood lactate (7.0 to 5.5 mmol/L for L1; Table 7). Thus, while blood pH changes were not directly monitored during the present study, the cardiorespiratory changes observed with ANS supplementation were consistent with prior investigations of NaHCO_3 _supplementation on VO_2 _kinetics. This observation appears to support the claim by the ANS manufacturer that regular use of this supplement can enhance metabolic buffering capacity and lower blood lactate responses during high intensity, submaximal exercise. Of course, further testing should be performed to directly evaluate this claim.

### UBP10 Test

The UBP10 test was administered as three successive trials with the first serving as a practice and the last two performed maximally. Following the 7-day loading phase, both groups increased mean W10 values, but only the treatment group's post-testing values increased significantly relative to pre-testing values (229 to 243 W; Table 2). However, neither cardiorespiratory nor blood lactate measures changed significantly for either group. Additionally, pre- to post-change in W10 values (Figure 2) showed that most subjects within *both groups *actually increased W10 from pre- to post-testing (9 of 12 for placebo group and 11 or 12 for treatment group).

There are several factors that may have contributed to the UBP10 tests lack of complete consistency with those from the constant-power and UBP60 tests. First, given that each of these tests required only 10-secs of maximal effort followed by 2.5 mins of complete rest between each trial, significant pre- to post-changes related to the UBP10 tests were not necessarily expected. However, for the sake of consistency, we chose to administer the UBP10 and UBP60 tests in the same manner as that described for the original development and validation of these tests [6]. In addition, pilot testing (prior to this study) with a protocol that required eight successive UBP10 tests with 30-sec rest intervals suggested that both peak HR and W10 were responsive to a 7-day ANS loading phase. Thus, the choice to be consistent with previous testing protocols, in addition to the brevity of the actual test time (10 seconds) and length of total rest (2.5 mins), probably contributed to the lack of meaningful cardiorespiratory or blood lactate changes in the treatment group. A second contributing factor is highlighted by the graphs of pre- to post-change in W10 (Figure 2). Close evaluation of these graphs indicate that most subjects increased the W10 regardless of group assignment. Thus, despite the previous evaluation of UBP10 reliability described in the Methods section, it seems likely that the UBP10 test was more skill dependent than the UBP60 test. This also suggests that the single familiarization visit was not sufficient for all subjects to achieve repeatable W10 values with successive visits.

### UBP60 Test

The UBP60 test, the last of the three UBP tests administered, required skiers to maintain the highest average UBP over the course of 60 seconds of double-poling. Interestingly, not only did peak values for HR (177 versus 184 BPM; Table 4), VO_2 _(3.26 versus 3.43 L/min; Table 5), and minute ventilation (V_E _- 153.3 versus 163.5 L/min; Table 6) all decreased significantly for post-testing in the treatment group, but the same group also generated more UBP following the 7-day loading phase (190 to 198 W for W60; Table 3). In addition, the last two post-testing recovery blood lactate measures (L7 and L8) for the UBP60 tests were significantly lower for the treatment group. In contrast, the placebo group showed no change in W60, peak HR, or peak V_E _while also showing significant increases in peak VO_2 _(Table 5) and the final recovery blood lactate (L8; Table 7) following the placebo group's 7-day loading period. Collectively, these observations suggest that the treatment group experienced less cardiorespiratory stress and lower recovery blood lactate values while generating more average power during post-testing.

In contrast to the individual changes in W10 between pre- and post-testing (Figure 2), the individual changes in W60 (Figure 3) showed that all treatment group subjects increased W60 from pre- to post-testing while the placebo groups' responses were highly variable. Again, in combination with the significant changes in cardiorespiratory and recovery blood lactate measures, the treatment groups' post-testing responses to the ANS loading suggests possible ergogenic benefits.

Given that the UBP60 test was the last of three tests administered, as well as the 60-sec test time for testing, the UBP60 test was though apriori to be most sensitive to creating significant cardiorespiratory and blood lactate changes following the ANS loading. Numerous studies investigating the influence of NaHCO_3 _supplementation on indicators of performance have used 30-120 sec time intervals for testing, as well as repeat test intervals following fixed rest intervals, to emphasize the use of non-mitochondrial ATP production and subsequent intracellular acidosis (for a review see Williams [14]). Thus, whether acidosis was induced acutely by the 60-sec time frame of the UBP60 test, or by the accumulation of three tests administered successively, or by a combination of these factors, the influence of ANS loading on the treatment group was similar to what has been described for NaHCO_3 _supplementation. Specifically, the treatment group was capable of generating higher W60 values while experiencing lower cardiorespiratory stress and lower recovery blood lactate values. These observations may support the claims by the ANS manufacturer of a more rapid recovery of muscle function following prior intense muscular efforts.

### Possible mechanism for observed effects?

The Alka-Myte^®^-based supplement evaluated by this study is purported to be a mineral-based intracellular and extracellular alkalizing agent that helps minimize the influence of metabolic acidosis and muscle fatigue during high intensity exercise. Classically, this type of buffering agent refers to mitigating the impact of excess intramuscular lactic acid on decreased intracellular pH and the subsequent performance decrement of cross-bridge cycling and muscle force generation [4,5]. However, the lactic acid hypothesis as a driving force behind metabolic acidosis and muscle fatigue is not supported by the current body of research [4,5]. The creation of metabolic acidosis during high intensity exercise has been shown to occur when the rate of ATP hydrolysis (i.e., an indicator of ATP demand) exceeds the rate of ATP production by the mitochondria [4]. As such, the formation of cytosolic lactic acid from pyrurate is actually caused by an increased cytosolic H^+ ^concentrations rather than lactic acid being the cause of increased H^+ ^concentrations. Thus, despite the frequent confusion in research and lay-literature regarding the primary cause of metabolic acidosis, measures of blood lactate during and immediately following exercise are still considered reasonable correlates of intracellular changes in pH for whole-body exercise [4].

Despite the lack of support for the lactic acid hypothesis, there is general agreement that metabolic acidosis can adversely influence muscle function [5]. Thus, any nutrition supplement that can potentially dampen the onset or severity of metabolic acidosis during high intensity exercise can also potentially influence muscle function and thus whole-body performance. For example, dosing with NaHCO_3 _[15,16], sodium citrate [1,16], or sodium lactate [16] have all been shown to positively influence physical performance. One likely mechanism by which these supplements influence metabolic acidosis is by improved intracellular and/or extracellular buffering of H^+^. However, since extracellular (i.e. plasma) acidosis will not occur until minutes after a bout of high intensity exercise, it is possible that improved extracellular buffering acts to increase the intra- to extracellular H^+ ^gradient during exercise [17]. In the present study, neither arterial blood samples nor muscle biopsy samples were collected for the measurement of pH or buffering capacity so we cannot be certain to what degree that the Alka-Myte^®^-based supplement was acting as an intra- or extracellular alkalizing agent. Future experimentation with this supplement should incorporate these measures to address this limitation.

One hypothesized mechanism relating the influence of intracellular metabolic acidosis on muscle fatigue is a postulated influence on the central nervous systems' ability to recruit the affected muscle fibers [5]. For example, Street et al. [17] has shown that extracellular alkalosis induced by sodium citrate ingestion will influence the accumulation of interstitial H^+^, which, in turn, was coupled to an increase in potassium ions (K^+^). Since the accumulation of interstitial K^+ ^has been shown to reduce muscle excitability [18], the lowering interstitial K^+ ^has also been postulated to improve performance of the affected muscle [19]. It has also been suggested that local pH and concentrations of K^+ ^are related to local vasodilatory mechanisms [20]. In short, induced extracellular alkalosis may influence blood flow indirectly through an influence on interstitial K^+ ^concentrations.

### Study limitations

As a pilot evaluation of this Alka-Myte^®^-based supplement, this study was designed simply to describe the effects of a proscribed supplementation routine rather than decipher possible mechanisms. Thus, future studies should verify the potential ergogenic effects of this supplement with more invasive measures of changes in blood pH. In addition, it is not known whether a 7-day loading phase was necessary for the observed treatment effects or whether a longer loading phase, or even a higher daily dosage, would elite different results. Thus, issues related to a dose-response paradigm must be addressed with future studies.

## Conclusions

In response to seven days of ingesting an Alka-Myte^®^-based alkalizing nutrition supplement, trained Nordic skiers experienced significant changes in cardiorespiratory, blood lactate, and upper body power output measures. All of the observed changes were consistent with those of an ergogenic aid for trained Nordic skiers. In contrast, a similar group of Nordic skiers consuming a placebo did not experience similar changes. Thus, the use of this supplement appeared to impart an ergogenic benefit to the skiers that may be similar to the effects expected from consuming well-studied extracellular buffering agents such as sodium bicarbonate.

## Competing interests

The authors acknowledge that the article-processing charge for this manuscript was paid by Rocktape (Los Gatos, CA USA). In addition, the tablets used for both treatment and placebo groups were provided without charge by TAMER Laboratories, Inc. (Shorline, WA USA).

## Authors' contributions

The primary author of this study was responsible for the study design, subject recruitment, data analysis, and manuscript preparation, while the remaining authors were responsible for health screening and data collection. All authors read and approved the final manuscript.

## References

[B1] Linossier MT Dormois D Bregere P Geyssant A Denis C Effect of sodium citrate on performance and metabolism of human skeletal muscle during supramaximal cycling exercise Eur J Appl Physiol 1997 76 48 54 10.1007/s004210050211 9243169

[B2] Potteiger JA Nickel GL Webster MJ Haub MD Palmer RJ Sodium citrate ingestion enhances 30 km cycling performance Int J Sports Med 1996 17 1 7 11 10.1055/s-2007-972800 8775569

[B3] Raymer GH Marsh GD Kowalchuk JM Thompson RT Metabolic effects of induced alkalosis during progressive forearm exercise to fatigue J Appl Physiol 2004 96 2050 2056 10.1152/japplphysiol.01261.2003 14766777

[B4] Robergs RA Ghiasvand F Parker D Biochemistry of exercise-induced metabolic acidosis Am J Physiol Regul Integr Comp Physiol 2004 287 R502 R516 10.1152/ajpregu.00114.2004 15308499

[B5] Cairns SP Lactic acid and exercise performance - culprit or friend? Sports Med 2006 36 4 279 291 10.2165/00007256-200636040-00001 16573355

[B6] Alsobrook NG Heil DP Upper body power as a determinant of classical cross-country skiing performance Eur J Appl Physiol 2009 105 4 633 641 10.1007/s00421-008-0943-z 19039602

[B7] Nilsson JE Holmberg HC Tveit P Hallen J Effects of 20-s and 180-s double poling interval training in cross-country skiers Eur J Appl Physiol 2004 92 121 127 10.1007/s00421-004-1042-4 15024663

[B8] Soper C Hume PA Reliability of power output during rowing changes with ergometer type and race distance Sports Biomech 2004 3 2 237 248 10.1080/14763140408522843 15552583

[B9] Pyne DB Boston T Martin DT Logan A Evaluation of the Lactate Pro blood lactate analyzer Eur J Appl Physiol 2000 82 112 116 10.1007/s004210050659 10879451

[B10] Cohen J Statistical Power Analysis for the Behavioral Sciences 1988 2 Hillsdale, NJ: Lawrence Erlbaum Associates

[B11] Berger NJA McNaughton LR Keatley S Wilkerson DP Jones AM Sodium bicarbonate ingestion alters the slow but not the fast phase of VO_2 _kinetics Med Sci Sports Exerc 2006 38 11 1909 1917 10.1249/01.mss.0000233791.85916.33 17095923

[B12] Kolkhort FW Rezende RS Levy SS Buono MJ Effects of sodium bicarbonate on VO_2 _kinetics during heavy exercise Med Sci Sports Med 2004 36 11 1895 1899 10.1249/01.MSS.0000145440.55346.28 15514504

[B13] Zoladz JA Szkutnik Z Duda K Majerczak J Korzeniewski B Preexercise metabolic alkalosis induced via bicarbonate ingestion accelerates VO_2 _kinetics at the onset of a high-power-output exercise in humans J Appl Physiol 2005 98 895 904 15516367 10.1152/japplphysiol.01194.2003

[B14] Williams MH Bicarbonate Loading Sports Sci Exchange 1992 36 4 1 4

[B15] Matson LG Tran ZV Effects of sodium bicarbonate ingestion on anaerobic performance: a meta-analytic review Int J Sport Nutr 1993 3 2 28 8388767 10.1123/ijsn.3.1.2

[B16] Van Montfoort MCE Van Dieren L Hopkins WG Shearman JP Effects of ingestion of bicarbonate, citrate, lactate, and chloride on sprint running Med Sci Sports Exerc 2004 36 7 1239 1243 10.1249/01.MSS.0000132378.73975.25 15235332

[B17] Street D Nielsen J-J Bangsbo J Juel C Metabolic alkalosis reduces exercise-induced acidosis and potassium accumulation in human skeletal muscle interstitium J Physiol 2005 566 2 481 489 10.1113/jphysiol.2005.086801 15860529 PMC1464741

[B18] Clausen T Na^+^K^+ ^pump regulation in skeletal muscle contractility Physiol Rev 2003 83 1269 1324 14506306 10.1152/physrev.00011.2003

[B19] Nielsen OB Ortenblad N Lamb GD Stephenson DG Excitability of the T-tubular system in rat skeletal muscle: roles of K^+ ^and Na^+ ^gradients and Na^+^-K^+ ^pump activity J Physiol 2004 557 133 146 10.1113/jphysiol.2003.059014 15034125 PMC1665049

[B20] Juel C Changes in interstitial K^+ ^and pH during exercise: implications for blood flow regulation Appl Physiol Nutr Metab 2007 32 846 851 10.1139/H07-065 18059608

